# Case Report: Inflammatory myofibroblastoma of the right forearm in an adult male: a clinical report

**DOI:** 10.3389/fonc.2026.1737213

**Published:** 2026-06-03

**Authors:** Lanlan Li, Xiang Chang, Zhishuai Hu, Min Wang, Xiaoguang Huo, Wenzhe Xu

**Affiliations:** 1 Zibo Central Hospital, Department of Rheumatology and Immunology, Shandong, China; 2 Zibo Central Hospital, Department of Medical Engineering, Shandong, China; 3 Zibo Central Hospital, Department of Vascular Surgery, Shandong, China; 4 Zibo Central Hospital, Department of Ultrasound, Shandong, China

**Keywords:** adult male, forearm, imaging diagnosis, inflammatory myofibroblastoma, treatment

## Abstract

**Background:**

Inflammatory myofibroblastoma (IMT) is rare, and cases located in the limbs are even rarer. We report a case of a 69-year-old Chinese male with a mass in the right forearm. It was surgically removed, and the pathology showed inflammatory myofibroblastoma. This case is reported due to its rare location. The purpose of this case report is to highlight the uniqueness of the case, collect and analyze the imaging characteristics of the disease, and provide assistance for daily clinical diagnosis and treatment.

**Case presentation:**

A 69-year-old male patient was reported with an inflammatory myofibroblastoma in the right forearm. A superficial ultrasound revealed a hypoechoic mass in the subcutaneous tissue of the right forearm. MRI showed a space-occupying lesion in the proximal subcutaneous area of the right forearm, and contrast-enhanced scanning was recommended for further evaluation. Postoperative pathological findings showed a malignant tumor of soft tissue origin in the right forearm, and immunohistochemistry suggested inflammatory myofibroblastoma.

**Conclusions:**

In summary, Inflammatory myofibroblastic tumor (IMT) in adults is a rare condition. When routine ultrasound or imaging detects limb masses, this disease should be considered.

## 1 Introduction

Inflammatory myofibroblastic tumor (IMT) is a rare mesenchymal tumor (1).

Initially considered a benign lesion, recent studies have shown it has malignant potential (2). The exact cause remains unknown, with pathological features including myofibroblast proliferation and extensive inflammatory cell infiltration. ALK protein expression is regarded as a representative immunological marker. It commonly occurs in the lungs but may also develop in abdominal, pelvic, or head and neck regions (3, 4). IMT lackstypical clinical symptoms and specificimaging manifestations. Diagnosis relies on comprehensive pathological and immunohistochemical analysis (5). Surgical resection remains the primary treatment (4, 6). The incidence rate is around 0.04%. This case report aims to provide a reference for clinicians by detailing the diagnosis and treatment of an extremely rare case of IMT in the right forearm. So far, the ultrasound report is extremely rare.

## 2 Case report

This study received approval from the Ethics Committee of Zibo Central Hospital, under ethics number 2025 Yan No. 082.

**Figure 1:**
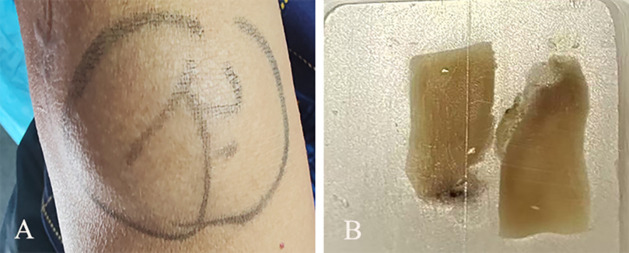
Surface view of the mass and postoperative section. **(A)** Surface view: a 3×3cm mass at the right elbow joint. **(B)** postoperative section: Partial tissue of the mass fixed in a paraffin block.

A 69-year-old male patient developed a mass near his right elbow about a year ago without apparent cause. Over the past four months, the mass had grown to approximately 3×3cm (**Figure 1A**). He was admitted to our hospital on January 6, 2025, with no significant tenderness or mobility restriction during the initial examination. No special treatment was administered externally, and he was admitted for surgical intervention.

**Figure 2:**
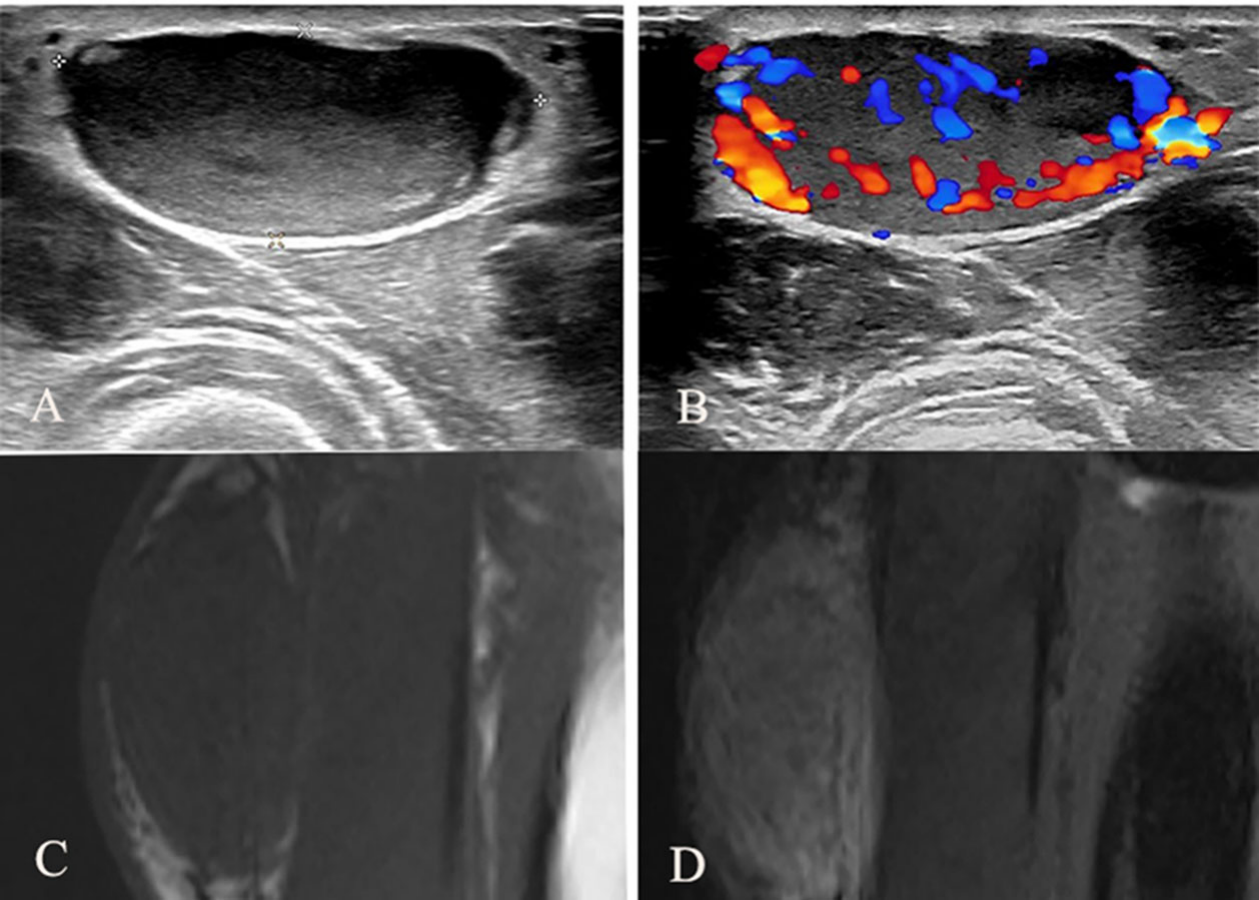
Ultrasound doppler and MRI of the mass: **(A)** 2D ultrasound shows a hypoechoic mass measuring approximately 34×15mm in the right forearm subcutaneous tissue, with well-defined borders and heterogeneous internal echogenicity. **(B)** CDFI reveals abundant blood flow signals. **(C)** T1WI weighted image demonstrates an oval-shaped abnormal signal with clear margins, measuring 1.7×3.7×3.2cm in size, showing relatively homogeneous internal signals with slightly hypointense characteristics. **(D)** T2WI fat-suppressed sequence displays slightly hyperintense signals.

Upon admission, the patient underwent a superficial mass ultrasound examination. The ultrasound revealed a hypoechoic mass measuring approximately 34×15mm in the right forearm subcutaneous tissue (**Figure 2A**), with well-defined borders and heterogeneous internal echogenicity. CDFI showed abundant blood flow signals within the mass (**Figure 2B**). The diagnosis was confirmed as a hypoechoic mass in the right forearm subcutaneous tissue.

To further characterize the lesion, we performed MRI of the superficial mass in the patient. The MRI revealed a well-demarcated, oval-shaped abnormal signal in the proximal lateral forearm subcutaneous tissue, measuring approximately 1.7×3.7×3.2cm in size. The internal signal showed homogeneous distribution with slightly prolonged T1 (**Figure 2C**) and T2 signals, and slightly hyperintense on T2WI fat-suppressed sequence (**Figure 2D**). The adjacent subcutaneous fat showed slightly hyperintense on PDWI. Diagnosis: Subcutaneous space-occupying lesion in the proximal right forearm. Contrast-enhanced scanning is recommended for further evaluation.

Laboratory tests showed that the patient’s nutritional status, metabolic health, blood routine, coagulation status and infectious disease indicators were within the normal range.

**Figure 3:**
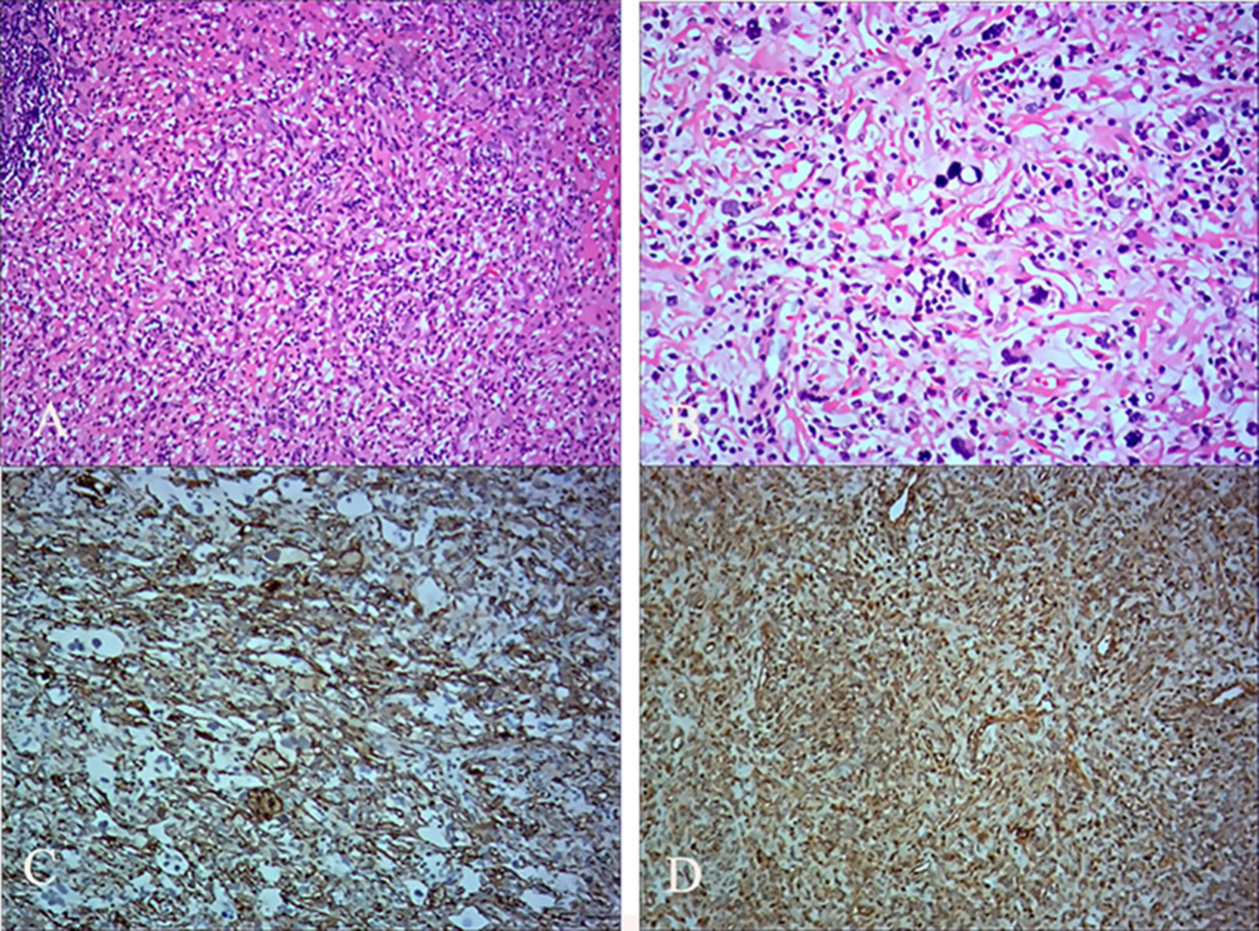
Histopathological and immunohisto-chemical findings. **(A)** Histopathological image (10×10); **(B)** Histopathological image (10×20); **(C)** Immunohisto-chemical staining for SMA (+) (10×10); **(D)** Immunohistochemical staining for Vimentin (+) (10×10).

Finally, the patient underwent a right forearm mass excision with peripheral nerve decompression under plexus anesthetization. A 7cm lateral incision was made along the anteromedial aspect of the right elbow, and the tissue was dissected layer by layer to the superficial muscle layer. A 3×3cm mass with a capsule containing “flesh-like” tissue was identified, presenting as soft with well-demarcated borders. The mass encased the right forearm cutaneous nerve, which was carefully dissected and the mass completely removed. Surrounding fascia and soft tissues were cleared. Postoperative pathology revealed a soft tissue-derived malignant tumor in the right forearm (**Figure 1B**). Immunohistochemistry indicated an inflammatory myofibroblastoma (tumor V: 3.5×3×2 cm), with no tumor invasion in the stromal nerve fiber bundles, no tumor thrombi in the vessels, and no normal tissue or tumor cells in the partial margins or free fat tissue. Immunohistochemistry results: Vimentin (+); S-100 scattered (+); SMA (+); desmin (-); STAT6 (-); CD68 (+); CDK4 cytoplasmic (+); MDM2 (-); β-catenin cytoplasmic (+); SOX10 (-); CD31/CD34 vascular (+); ERG diffuse (+); EMA focal (+); CD20/CD3 focal lymphocyte clusters (+); P53 focal (+); Ki-67 (+) with 45% tumor stromal cells (**Figure 3**).

## 3 Discussion

Inflammatory myofibroblastoma is a rare disease first reported in 1939 as occurring in the lung or pleura4. In 1954, Umiker and Iverson named it inflammatory pseudotumor (7). Most scholars previously considered inflammatory pseudotumor a benign tumor. However, recent studies have revealed that some inflammatory pseudotumors may exhibit potential malignant tendencies (8). In 2002, the WHO officially designated this condition as IMT (9), classifying it as a mesenchymal tumor with low-grade malignancy or borderline characteristics (10).

The etiology and pathogenesis of IMT remain unclear (11). The case we report lacks specific medical history.

Since this disease is rare, literature reports that it mainly occurs in children and young adults (12). The median age was around 10 years old.The case we reported was a 68-year-old man.

Only one case of IMT in the upper limb has been reported, involving the brachial artery in the upper arm (9, 13). The forearm IMT case we report is extremely rare and has not been documented previously. The clinical manifestations of IMT are nonspecific (14). The signs and symptoms of IMT are related to the mass effect of the tumor and local inflammation.It varies according to the different anatomical locations. Patients may present with fever and weight loss. Tumors in the upper and lower respiratory tracts may cause pain, dyspnea, airway obstruction, epistaxis, and headache; tumors in the neck may cause hoarseness; tumors in the digestive tract may lead to intestinal obstruction and constipation; tumors in the liver may cause jaundice andhepatosplenomegaly; tumors in the urinary tract may cause dysuria and hematuria; tumors in the uterus may cause abnormal bleeding in the reproductive tract. In terms of laboratory tests, patients with IMT often show elevated inflammatory indicators, such as increased C-reactive protein levels, accelerated erythrocyte sedimentation rate, and in blood routine examination, there may be elevated white blood cells, anemia, and increased platelets.

In this case, the patient showed no tenderness, limited mobility, or significant clinical symptoms.

Before pathological diagnosis, imaging assessment is often an important step in clinical detection of lesions, determination of nature and planning of treatment strategies. Although computed tomography and magnetic resonance imaging can provide good anatomical structure display, which is particularly suitable for evaluating the relationship between the extent of tumor invasion and adjacent organs, their sensitivity and specificity are limited in the early identification of IMT, and the cost is relatively high. Some patients have contraindications to contrast agents. In contrast, ultrasound examination has the advantages of non-invasiveness, repeatability, low cost and real-time dynamic observation, and has broad application potential in the preliminary assessment of superficial or superficial soft tissue lesions.

Color Doppler flow imaging was used to further analyze the blood flow conditions inside and around the lesions and the characteristics of blood flow spectra. Grading of blood flow signals: Grade 0, no obvious blood flow signals in the lesions; Grade I, only 1–2 punctate or short strip-like blood flow signals are seen; Grade II, multiple blood flow signals can be seen in the lesions, but the distribution is still limited; Grade III, rich blood flow signals in the lesions or showing a penetrating distribution.In the preoperative ultrasound findings, the lesion morphology, size, and degree of blood flow perfusion in IMT patients are related to the potential malignancy. Among them, blood flow grading has important indicative value. Grade III blood flow perfusion is only seen in patients with a malignant tendency, and benign types all show blood flow of grade 0 - I. If rich internal blood flow is shown under color Doppler ultrasound, accompanied by an arterial spectral waveform, it also indicates a high biological activity and the possibility of local invasion, and the alertness to the malignant risk should be increased.

IMT ultrasound typically reveals an isolated hypoechoic mass with well-defined borders, regular shape, and homogeneous internal echogenicity, showing abundant blood flow signals on CDFI (11, 15). The findings in this case align with these characteristics. In imaging studies, most pulmonary IMT lesions appear as well-defined solid masses on CT scans (13). CT imaging often demonstrates IMT as uniformly enhanced lesions with moderate to strong intensity. On MRI, IMT appears as a solid tumor with low or isointense signal intensity on T2-weighted images, showing uniform contrast enhancement. T1-weighted imaging reveals homogeneous low signal intensity with post-contrast enhancement, while fat-suppressed T2 sequences demonstrate high signal intensity (11), consistent with this case. Although imaging modalities like IMT and ultrasound cannot confirm diagnosis, they provide valuable diagnostic clues for differential evaluation.

The definitive diagnosis of IMT primarily relies on postoperative pathological examination. During IMT surgery, the tumor typically presents as a solid mass with one or multiple nodules, usually of considerable size (16). Histologically, IMT is predominantly composed of spindle-shaped cells, accompanied by varying amounts of inflammatory components such as lymphocytes and plasma cells, along with collagen. The presence of chronic inflammatory cells, including lymphocytes and plasma cells, in the stroma is a hallmark of this pathology (17). Immunohistochemical staining showing positive expression of SAM and Vimentin serves as the definitive diagnostic criterion for IMT (18).The pathological diagnosis of IMT needs to be differentiated from a variety of spindle cell tumors and reactive proliferative diseases, such as nodular fasciitis, gastrointestinal stromal tumor, follicular dendritic cell tumor, inflammatory fibroid polyp, IgG4-related disease, etc.

Surgical operation remains the preferred method for treating localized IMT (19). Studies have shown that the prognosis of completely resected IMT is good, and the 5-year survival rate is 91% (20).In some patients, the tumor recurs after surgical treatment. After complete resection, the recurrence rate of IMT may vary depending on the location of the lesion. The recurrence rate of lesions in the lung is 2%, while that of extra-pulmonary lesions can reach 25%.

For patients with postoperative recurrence, radiotherapy may provide partial remission (21).Due to the extremely low incidence of IMT, the literature mainly consists of case reports. The tumor locations cover the skull base, head and neck, chest, etc. The recommended radiation dose is mostly 45 ± 60 Gy, often combined with glucocorticoids or other immunosuppressive agents. The follow-up period varies from 2 to 7 years, and the 5-year survival rate is over 70% (22).

In cases of unresectable lesions, combination therapy with anti-inflammatory drugs and corticosteroids has shown success in managing IMT (12). A documented case reported tumor shrinkage and over a year of recurrence-free survival in a patient with IMT in the ribs and intercostal muscles treated solely with oral steroids (17). The ALK gene may activate the tyrosine kinase pathway, contributing to local recurrence and metastasis of tumors (23). Patients with positive ALK gene testing may benefit from ALK inhibitors (24). In addition to ALK targeted therapy, there is also targeted therapy for other fusion genes.RET fusions have been identified in ALK-negative IMT patients (25). Selpercatinib is approved in the United States for the treatment of RET fusion solid tumors. However, there is currently limited data on targeted therapy for other targets in IMT, and further research is needed.69% of tumors in IMT showed positive PD-L1 expression (26). Reports on the treatment of IMT patients with immune checkpoint inhibitors are very limited.One case of nasopharyngeal IMT patient achieved partial remission after using toripalimab and sintilimab (27).Further exploration and experience accumulation are still needed for the immunotherapy of IMT.

Whole body treatment is applicable to patients with advanced and inoperable conditions. Currently, due to the rarity of the disease and the lack of prospective data on the efficacy of chemotherapy, no standard chemotherapy regimen has been established for advanced IMT. Retrospective studies (28). have shown that the objective response rate (ORR) of chemotherapy regimens with anthracyclines or methotrexate ± vinorelbine/vinblastine is 50%. There are also reports (29, 30) suggesting that pemetrexed + platinum and paclitaxel + platinum have good efficacy for IMT. It is recommended to organize clinical trials of IMT chemotherapy regimens as much as possible to determine the efficacy of chemotherapy regimens.

There is no consensus on the most effective non-surgical approach (17). In this case, surgical resection achieved satisfactory outcomes, with follow-up examinations showing no recurrence.

## 4 Conclusion

Inflammatory myofibroblastoma (IMT), a rare mesenchymal tumor, is challenging to diagnose preoperatively, with surgery being the primary treatment.preoperative ultrasound has certain reference value in evaluating the potential malignant risk of IMT. Irregular lesion morphology, unclear boundaries, obvious blood perfusion (especially grade III), and larger tumor diameter may indicate a higher malignant tendency. Combining imaging and immunohistochemical results helps with preoperative risk stratification and clinical treatment decision-making. Given its high recurrence rate, long-term follow-up is essential. This case report and literature review aim to enhance clinicians’ understanding of IMT and provide valuable insights for clinical evaluation and management.

## Data Availability

The original contributions presented in the study are included in the article/supplementary material. Further inquiries can be directed to the corresponding author.

## References

[1] Nakano K . Inflammatory myofibroblastic tumors: recent progress and future of targeted therapy. Jpn J Clin Oncol. (2023) 53:885–92. doi: 10.1093/jjco/hyad074. PMID: 37394916

[2] Rich BS Fishbein J Lautz T Rubalcava NS Kartal T Newman E . Inflammatory myofibroblastic tumor: a multi-institutional study from the Pediatric Surgical Oncology Research Collaborative. Int J Cancer. (2022) 151:1059–67. doi: 10.1200/jco.2021.39.15_suppl.10024. PMID: 35604778

[3] Yang H Lai B . Inflammatory myofibroblastoma mimicking cavernous hemangioma in the liver. Liver International: Off J Int Assoc For Study Liver. (2024) 44:1265–6. doi: 10.1111/liv.15884. PMID: 38407558

[4] Qian X Ning W Dunmall LC Qu Y Wang Y Zhang H . Treatment of intracranial inflammatory myofibroblastic tumor with PD-L1 inhibitor and novel oncolytic adenovirus Ad-TD-nsIL12: a case report and literature review. Front Immunol. (2024) 15:1427554. doi: 10.3389/fimmu.2024.1427554. PMID: 39114662 PMC11303231

[5] Wachter F Al-Ibraheemi A Trissal MC Hollowell M DuBois SG Collins NB . Molecular characterization of inflammatory tumors facilitates initiation of effective therapy. Pediatrics. (2021) 148. doi: 10.1542/peds.2021-050990. PMID: 34814185

[6] Crisan D Wortsman X Catalano O Badea R Kastler S Badea A . Pre-operative high-frequency ultrasound: a reliable management tool in auricular and nasal non-melanoma skin cancer. J der Deutschen Dermatol Gesellschaft J German Soc Dermatol: JDDG. (2024) 22:357–65. doi: 10.1111/ddg.15308. PMID: 38243870

[7] Umiker WO Iverson L . Postinflammatory tumors of the lung; report of four cases simulating xanthoma, fibroma, or plasma cell tumor. J Thorac Surg. (1954) 28:55–63. 13175281

[8] Xie Y Tang W Ma J Wang Y Chen Y . Elevated 68 Ga-FAPI activity in pulmonary inflammatory pseudotumor. Clin Nucl Med. (2024) 49:777–8. doi: 10.1097/rlu.0000000000005276. PMID: 38768090

[9] Singh G Kumar A J AC Jaiswal S Verma P Mehrotra A . Inflammatory myofibroblastic tumor in the intradural extramedullary space of the lumbosacral spine: a case report and review of the literature. Child's Nervous System: ChNS: Off J Int Soc For Pediatr Neurosurg. (2024) 41:24. doi: 10.1007/s00381-024-06690-4. PMID: 39615002

[10] Lin J Liu H Zhuang Y Yang P Zheng Y Yang Y . Inflammatory myofibroblastic tumor of the thigh without bone involvement: a case report. World J Surg Oncol. (2014) 12:208. doi: 10.1186/1477-7819-12-208. PMID: 25022487 PMC4114126

[11] Yuan C Fan J Xu L . Inflammatory myofibroblastic tumor of the upper arm: a case report. Medicine. (2023) 102:e36558. doi: 10.1097/md.0000000000036558. PMID: 38115338 PMC10727551

[12] Hamdouni W Bellalah A Bchir S Alaoui I Abdelaaly M Ktari K . A giant ureteral inflammatory myofibroblastic tumor in a 4-year-old child: a case report and review of the literature. Urology. (2022) 165:312–5. doi: 10.1016/j.urology.2022.01.028. PMID: 35101546

[13] Si X Wu S Feng R Wang H Zhang X Zhang L . Clinicopathological characteristics of inflammatory myofibroblastic tumor: a single center retrospective cohort study. Thorac Cancer. (2025) 16:e15496. doi: 10.1111/1759-7714.15496. PMID: 39592917 PMC11729751

[14] Coffin CM Watterson J Priest JR Dehner LP . Extrapulmonary inflammatory myofibroblastic tumor (inflammatory pseudotumor). a clinicopathologic and immunohistochemical study of 84 cases. Am J Surg Pathol. (1995) 19:859–72. doi: 10.1097/01.pas.0000213393.57322.c7. PMID: 7611533

[15] Raad RA Haddad L Jabbour T El-Rassi Z . Inflammatory myofibroblastic tumor of the liver mimicking metastasis on 18F-FDG PET/CT. Clin Nucl Med. (2021) 46:47–8. doi: 10.1097/rlu.0000000000003356. PMID: 33156048

[16] Coffin CM Patel A Perkins S Elenitoba-Johnson KS Perlman E Griffin CA . ALK1 and p80 expression and chromosomal rearrangements involving 2p23 in inflammatory myofibroblastic tumor. Modern Pathol: Off J United States Can Acad Pathol Inc. (2001) 14:569–76. doi: 10.1038/modpathol.3880352. PMID: 11406658

[17] Watanabe R Ano S Kikuchi N Saegusa M Shigemasa R Kondo Y . Inflammatory myofibroblastic tumor directly invading the right first rib treated with oral steroids: a case report. BMC Pulmonary Med. (2024) 24:67. doi: 10.1186/s12890-024-02873-6. PMID: 38308319 PMC10835977

[18] Ge R Liu C Yin X Chen J Zhou X Huang C . Clinicopathologic characteristics of inflammatory pseudotumor-like follicular dendritic cell sarcoma. Int J Clin Exp Path. (2014) 7:2421–9. PMC4069939 24966952

[19] Song X Lou S Han Y Yang C . Inflammatory myofibroblastic tumor of the thigh: a case report. Asian J Surg. (2024) 47:791–2. doi: 10.1016/j.asjsur.2023.10.044. PMID: 37879995

[20] Sagar AES Jimenez CA Shannon VR . Clinical and histopathologic correlates and management strategies for inflammatory myofibroblastic tumor of the lung. a case series and review of the literature. Med Oncol (Northwood London England). (2018) 35:102. doi: 10.1007/s12032-018-1161-0. PMID: 29869302

[21] Sun L Tu L Wang X Zhu H Mao J Zhuo H . Management of rectal inflammatory myofibroblastic tumor recurrence. J Cancer Res Ther. (2014) 10:425–7. doi: 10.4103/0973-1482.136679. PMID: 25022414

[22] Zhu Z Zha Y Wang W Wang X Gao Y Lv W . Inflammatory myofibroblastic tumors in paranasal sinus and nasopharynx: a clinical retrospective study of 13 cases. BioMed Res Int. (2018) 2018:7928241. doi: 10.1155/2018/7928241. PMID: 30410939 PMC6205320

[23] Pecoraro Y Diso D Anile M Russo E Patella M Venuta F . Primary inflammatory myofibroblastic tumor of the trachea. Respirol Case Rep. (2014) 2:147–9. doi: 10.1002/rcr2.81. PMID: 25530866 PMC4263498

[24] Yi ES Chung JH Kulig K Kerr KM . Detection of anaplastic lymphoma kinase (ALK) gene rearrangement in non-small cell lung cancer and related issues in ALK inhibitor therapy: a literature review. Mol Diagn Ther. (2012) 16:143–50. doi: 10.1007/bf03262202. PMID: 22506598

[25] Cheek EH Fadra N Jackson RA Davila JI Sukov WR Uckerman MT . Uterine inflammatory myofibroblastic tumors in pregnant women with and without involvement of the placenta: a study of 6 cases with identification of a novel timp3-ret fusion. Hum Pathol. (2020) 97:29–39. doi: 10.1016/j.humpath.2019.12.006. PMID: 31917155

[26] Cottrell TR Duong AT Gocke CD Xu H Ogurtsova A Taube JM . Pd-l1 expression in inflammatory myofibroblastic tumors. Modern Pathol: Off J United States Can Acad Pathol Inc. (2018) 31:1155–63. doi: 10.1038/s41379-018-0034-6. PMID: 29449680 PMC6076347

[27] Meng X Zhang L Wang Q Chen J Zhang C Tao R . Genetic testing and immunotherapy for intracranial inflammatory myofibroblastic tumor: a case report. Onco Targets. (2022) 15:313–21. doi: 10.2147/ott.s343562. PMID: 35401006 PMC8985701

[28] Baldi GG Brahmi M Lo Vullo S Cojocaru E Mir O Casanova M . The activity of chemotherapy in inflammatory myofibroblastic tumors: a multicenter, european retrospective case series analysis. Oncologist. (2020) 25:e1777-e84. doi: 10.1634/theoncologist.2020-0352. PMID: 32584482 PMC7648357

[29] Si X Wang H Zhang X Wang M You Y Zhang L . Successful treatment of pulmonary inflammatory myofibroblastic tumor with platinum-pemetrexed: the first report of two cases. Oracic Cancer. (2020) 11:2339–42. doi: 10.1111/1759-7714.13520. PMID: 32495499 PMC7396391

[30] Kubo N Harada T Anai S Otsubo K Yoneshima Y Ijichi K . Carboplatin plus paclitaxel in the successful treatment of advanced inflammatory myofibroblastic tumor. Internal Med (Tokyo Japan). (2012) 51:2399–401. doi: 10.2169/internalmedicine.51.7599. PMID: 22975556

